# Co-creation or Co-destruction: A Perspective of Online Customer Engagement Valence

**DOI:** 10.3389/fpsyg.2020.591753

**Published:** 2021-02-03

**Authors:** Junaid Siddique, Amjad Shamim, Muhammad Nawaz, Ibrahima Faye, Mobashar Rehman

**Affiliations:** ^1^Department of Management and Humanities, Universiti Teknologi PETRONAS, Seri Iskandar, Malaysia; ^2^Department of Humanities, COMSATS University Islamabad, Islamabad, Pakistan; ^3^Department of Fundamental and Applied Sciences, Universiti Teknologi PETRONAS, Seri Iskandar, Malaysia; ^4^Faculty of Information and Communication Technology, Universiti Tunku Abdul Rahman, Kampar, Malaysia

**Keywords:** service co-destruction, service co-creation, online shopping, valence, engagement (involvement)

## Abstract

The increasing interest in online shopping in recent years has increased the importance of understanding customer engagement valence (CEV) in a virtual service network. There is yet a comprehensive explanation of the CEV concept, particularly its impact on multi-actor networks such as web stores. Therefore, this study aims to fill this research gap. In this study, past literature in the marketing and consumer psychology field was critically reviewed to understand the concept of CEV in online shopping, and the propositional-based style was employed to conceptualize the CEV within the online shopping (web stores) context. The outcomes demonstrate that the valence of customer engagement is dependent on the cognitive interpretation of signals that are prompted by multiple actors on a web store service network. If the signals are positively interpreted, positive outcomes such as service co-creation are expected, but if they are negatively interpreted, negative outcomes such as service co-destruction are predicted. These notions create avenues for future empirical research and practical implications.

## Introduction

The advancement in technology and the rise of industrial revolution 4.0 have increased the appeal of online shopping, and it can be observed through the dramatic increase of worldwide volume of online sales—USD 1,336 billion in 2014 to USD 2,382 billion in 2017, grows to USD 2,982 billion in 2018, and is expected to reach USD 5,695 billion by 2022 (Statista, 2019). The pattern demonstrates the promising prospects that online businesses have in the upcoming years. Furthermore, renowned web stores such as Tencent, Aliba, and eBay are examples of conventional business engagement with online business models, consequently providing significant benefits for firms, and customers.

Online shopping provides customers with the option to evaluate brands based on comments and ratings (Ozen and Engizek, 2014) and enable them to save time, cost, and energy. Through online shopping, businesses can reduce operational costs and resource expenditure, allowing a bigger customer database to be generated. In addition, businesses can communicate with the customers virtually, ensuring positively engaged customers for a competitive edge (Kumar and Pansari, 2016). Engagement is the customers’ psychological state of mind resulting from the interaction with the web stores and its associated services during the process of purchase (Van Doorn et al., 2010), and understanding its impact is crucial in building a customer-centric business model.

Customers’ engagement with a web store can be advantageous for businesses (Thakur, 2018) as it can lead to actual purchases (Brien and Toms, 2010). Following this, web stores tend to provide esthetically pleasant, user-friendly, and secure web store platforms to provide customers with a unique shopping experience that may lead to actual purchases (Lăzăroiu et al., 2020). According to the signaling theory, the situation is symmetric information exchange between the online service provider and the customer, which generates positive outcomes (Connelly et al., 2011) as well as a negative outcome, especially if the customers experienced or witnessed a bad shopping experience. The customers’ engagement with a brand, product, service, and web store that is based on their experience, be it negative or positive, is referred to as customer engagement valence (CEV; Van Doorn et al., 2010).

There have been several debates on the development of CEV and its possible outcomes (Storbacka et al., 2016; Li et al., 2017, 2018). Some argued that engagement is context-specific (Brodie et al., 2011) where customers either positively or negatively engaged depending on the context (Hollebeek and Chen, 2014; Juric et al., 2015). Positive engagement will yield positive outcomes such as purchase behavior, satisfaction, and loyalty (Hollebeek and Chen, 2014), but negative engagement may spread negative reviews (Loureiro and Kaufmann, 2018) that ward off customers (Weisstein et al., 2017). Nonetheless, some prove that negative engagement may cause positive outcomes (Juric et al., 2015), repeat purchase intention, and loyalty (Bruneau et al., 2018). These contradictions demonstrate that a clear line of inquiry between antecedents and CEV outcomes is missing.

In this study, the service-dominant logic (SDL) lens was used to assess the nature of CEV in the online shopping context. In online shopping, various actors trade ideas and experiences to co-create service (Vargo and Lusch, 2016; González-Torres et al., 2020). In the signaling theory perspective, the information shared for co-creation of service act as nodes in the service eco-system (Pee et al., 2018). The information nodes prompt customers to engage with various actors on the platform. This study intends to identify the possible outcomes on the customers’ engagement in which the web store utilizes information nodes as a medium of information.

Moreover, based on the integration of thoughts and SDL perspective, this study proposes that CEV in a web store can cause service co-creation or service co-destruction. The CEV is taken as a psychological, cognitive, and interactive component, in which the direction of customers’ engagement depends on their cognitive interpretation of the signals transmitted by other actors in a virtual network. Customers tend to engage positively with a web store if they have a positive and pleasant view of the web store (Vivek et al., 2012), especially if they have the opportunity to collaborate with other customers (Grönroos and Voima, 2013). The collaboration is called collaborative service co-creation (Bhalla, 2010). In contrast, unpleasant and negative perceptions for the web store will result in negative engagement (Juric et al., 2015; Naumann et al., 2017), causing service co-destruction through negative comments and low ratings. This study proposed that CEV is context-specific; it can generate service co-creation or service co-destruction based on information available on the web store and customers’ experience.

## Literature Review

### Customer Engagement in Online Shopping

Customer engagement (CE) is a wide niche in marketing research (Kumar et al., 2010; Brodie et al., 2011; Vivek et al., 2012; Moliner et al., 2018), and it pertains to the customers’ psychological state of mind that is induced by their experience in the engagement with the objects (such as web stores or actors). Engagement is a multidimensional construct composed of the cognitive, emotional, and behavioral states (Brodie et al., 2011) that can be observed through participation and involvement (Gebauer et al., 2013), which includes behavioral indications such as word of mouth (Gebauer et al., 2013), and altruistic behaviors (Hsieh and Chang, 2016). Past studies have investigated engagement through multiple perspectives—CE behaviors (Van Doorn et al., 2010; Jaakkola and Alexander, 2014; Verleye et al., 2014), consumer engagement (Brodie et al., 2013), consumer brand engagement (Rana and Dwivedi, 2016; Solem and Pedersen, 2016), customer-brand community engagement (Gummerus et al., 2012), service technology engagement (Bolton and Saxena-Iyer, 2009), advertising engagement (Phillips and Mcquarrie, 2010), and brand engagement in self-concept (Sprott et al., 2009). Most of these studies are within the context of physical customers’ interaction, which is different from virtual CE; the latter involves multiple actors simultaneously (customer-to-web store engagement, customer-to-e-retailer engagement, and customer-to-CE). The multiplicity causes inconsistent outcomes of CE as each engagement provides an individual interaction experience, hence, assessing CEV is of higher importance compared to considering CE only.

### Customer Engagement Valence

Valence refers to the positive association with an individual’s behavior, emotions, evaluation, and cognition (Colombetti, 2005), and it was initially perceived to be formed from an object (Li et al., 2018). It is claimed that an object influences attraction (positive force) or repulsion (negative force); it influences the direction of behavior (Tolman, 1932). Apart from that, an individual’s evaluation, cognition, and emotions also influence valence (Dulabh et al., 2018) as positivity and negativity are related to the emotions of a person (Brodie et al., 2011; Li et al., 2018) and valence is the consequence of an individual’s evaluation of the object (Hollebeek et al., 2014). In marketing research, valence is explored as CEV—a behavioral outcome of CE with an object, brand, or company’s resources. Hollebeek and Chen (2014) define CEV as the positive and negative engagement of customers associated with the brand’s favorable (positive) and unfavorable (negative) emotions, behavior, and thoughts. Bowden et al. (2015, 2017), and Naumann et al. (2017) echo this notion, claiming that the valence of customers’ engagement can be influenced by different attributes such as a behavioral outcome and the brand’s key attributes. Nonetheless, these studies focus only on the physical interaction settings. A brief on past studies on engagement valence is provided in Table 1.

**TABLE 1:** Past literature on engagement valence.

**S/No**	**Methodology**	**Source**	**Engagement valence**	**Research context**
1.	Conceptual	Van Doorn et al., 2010 Journal of service research	The valence of engagement perceived based on the positive and negative outcomes.	Theoretical foundations of customer engagement
2.	Exploratory	Hollebeek and Chen, 2014 Journal of product and brand management	Positive and negative outcomes of customer engagement refer to positive and negative outcomes.	The conceptual model for positive and negative customer engagement
3.	Exploratory	Bowden et al., 2015 Journal of marketing management	If the outcome of engagement is withdrawal, customer engagement is considered as positive and vice versa.	Engagement and disengagement of customers
4.	Conceptual	De Villiers, 2015 Journal of business research	The valence of engagement is perceived based on the behavioral outcome of the engagement.	New perspective in consumer brand engagement literature
5.	Exploratory	Bowden et al., 2017 Book chapter from customer engagement: Contemporary issues and challenges	The valence of engagement is perceived based on the behavioral outcome of the engagement.	Positive and negative engagement in online brand communities
6.	Exploratory	Juric et al., 2015 Book chapter from customer engagement: Contemporary issues and challenges	If a customer is positively engaged, then the behavioral outcomes are beneficial for others, and if the customer is negatively engaged, then the outcomes are harmful to others in a network	Negative engagement of customers in blogs.
7.	Exploratory	Dolan et al., 2017 Book chapter from customer engagement: Contemporary issues and challenges	Positive engagement in social media leads the participant to consume or contribute to user-created content. Negative engagement leads the participant to withdraw or to contribute negatively to user-created contents.	Social media engagement
11.	Exploratory	Li et al., 2018 Journal of service theory and practice	The outcome of engagement can be positive or negative, which is perceived differently by different actors in a network.	Engagement valence of actors in a network
12.	Conceptual	Li et al., 2017 Journal of service management	The valence of engagement resides in the past, present, and future psychological disposition that shifts between positive, negative, and ambivalent engagement	Multi-actor engagement in a network

There is limited research on CEV within virtual environments such as a web store, an interactive platform that provides various cues (signals) to the customers (Wells et al., 2011). Ease of use, brand and warranty information, product features, retailer ratings, delivering options, and price comparisons are some of the signals often used by the customers in their decision-making process while shopping online (Wells et al., 2011); hence, they are important in assessing customer’s level of engagement (Connelly et al., 2011). In the process of online shopping, an actor (web store management) communicates information (signals) that is interpreted and engaged by another actor (customer), who then provides feedback (comments or purchase) to the initial actor. The engagement takes place virtually, and web store management does not have control over the engagement valence on the receiving end as it relies entirely on the customer’s understanding and interpretation of the information. The valence is also influenced by information shared by other actors, such as comments and ratings.

The customers are considered to be positively engaged if the information received by all nodes and actors is symmetric (Wells et al., 2011), and they are more likely to start co-creating the service with the web store and other actors. If the information is asymmetric, customers might engage negatively with a web store, resulting in service co-destruction.

### Service Co-creation and Co-destruction

Service is the use of knowledge and skills to produce real value (value-in-use) from the potential values of products (Grönroos, 2011) and is co-created through the collaboration of multiple actors and resources integration with the aim to create value for the benefit of all (Vargo and Lusch, 2016; Shamim et al., 2017). Co-creation cannot occur without the engagement of multiple actors (Shamim et al., 2016). Resource integration is the exchange of service among the actors (Li et al., 2018). The co-creative service produces value for all parties—customers gain utilities from the products and services, and companies gain financial value, customer equity, customer trust, and customer satisfaction (Chandler and Lusch, 2015). Nevertheless, such dyadic interaction may cause negative engagement such as service failure, bad review comments, late delivery, or any other arising factors that negatively influence the customer’s behavior (Čaić et al., 2018).

Web stores provide a platform in which service providers are available to facilitate customers in experiencing unique value creation as the service providers are eager to provide services that can produce service co-creation instead of service co-destruction. Service platforms (web stores) mediate this process by facilitating the interaction between different actors, a crucial part of the service eco-system (Lusch and Nambisan, 2015). Web stores facilitate the interaction between actors such as web store personnel, suppliers, retailers, distributors, and customers for co-creating service, and are developed with different features and structures based on their product variety and design hierarchy (Kane and Bigham, 2014). For example, relationships on Facebook are initiated by a one-sided request and are based on friend requests, while Twitter operates based on followings (Kane and Bigham, 2014). In the same vein, web stores enable a business relationship between different actors to trade information, knowledge, and experience that are deemed beneficial for all actors (Vargo and Lusch, 2016). Positive interaction leads to co-creative service, while negative interaction will co-destruct the service.

## Conceptualization of CEV

**FIGURE 1:**
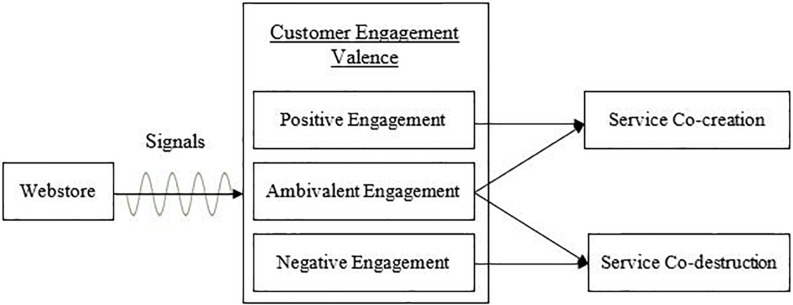
Engagement valence outcomes framework.

In this study, the signaling theory is used to conceptualize CEV and establish its relationship with service co-creation and service co-destruction pertaining to web store online shopping (Figure 1). Online business is a wide actor-to-actor service network composed of multiple actors and institutions that interact with each other and share institutions to co-create value. Signals (cues) such as information, web store esthetics, comments, and ratings are used to interact with each other and to engage with the customers who will later interpret the signals and behave accordingly (Kirmani and Rao, 2000). As mentioned before, engagement is dependent on the cognitive interpretation of signals generated by web stores. Based on SDL, different actors integrate their knowledge (comments and rating) in their own experience, and one customer’s knowledge may affect the purchase decision of other customers in a positive (service co-creation) or negative (service co-destruction) way (Floh et al., 2013). This study proposes that customers who interpret the signals positively will develop service co-creation, while those who interpret the signals negatively will develop service co-destruction.

## Propositions for CEV in Service Co-Creation Network

There are different conceptualizations of CE. A stream believes that valence is closely related to the behavior of customers during interaction with a brand or human—positive or negative behavior is caused by positive or negative engagement (Hollebeek and Chen, 2014; Bowden et al., 2015; Triantafillidou and Siomkos, 2018). Meanwhile, another stream believes that behavioral engagement and its outcome are independent of each other (Li et al., 2017) and that the valence is within the behavioral engagement, not its outcomes. As stated by Li et al. (2018), “the valence of actor engagement resides in the focal actor’s past, current and future psychological disposition.”

In online shopping, customers’ engagement is human–computer interaction. Past researches mainly focus on engagement in customer–brand or customer–customer interactions, which have different attraction or repulsion aspects. Different aspects pertain to human–computer interaction (Li et al., 2015), such as esthetics, ease of use, comments, and rating, which act as signals in prompting customers’ engagement and behavior (Wells et al., 2011; Zhang et al., 2017). A positive signal may be negative to another customer—for example, delivery time may not be of the same importance between different customers. Based on the signaling theory, this study proposes that:

*Proposition 1*: Valence of CE exists in the cognitive interpretation of signals sent by other customers and service providers in a web store.

Moreover, a customer can engage positively or negatively (Hollebeek and Chen, 2014), or they may have ambivalent emotions toward a web store (Li et al., 2018) as it can be both trusted and distrusted at the same time (Moody et al., 2014). Since the valence of engagement is associated with the cognitive interpretation of signals, the engagement may be ambivalent, and the valence can be positive, negative, or ambivalent (Li et al., 2018). The shift between the valences is worthy of being studied.

Actors in web stores utilize signals (information and knowledge sharing; Moody et al., 2014); positive engagement is caused by positive interpretation of the signals (Kirmani and Rao, 2000; Ullah et al., 2016). Furthermore, symmetric knowledge sharing and service exchange between different actors can produce positive engagement, asymmetric information may produce negative engagement, and simultaneous symmetric and asymmetric information exchange may lead to ambivalent engagement. Therefore, this study proposes that:

*Proposition 2*: Valence of CE shifts between positive, negative, and ambivalent based on the symmetric or asymmetric knowledge sharing or service exchange.

There is a lack of depth in the understanding of the conditions that affect the outcome of CEV. Hollebeek and Chen (2014) argues that that the outcomes are not based on the direction of engagement; it is the opposite as some negatively engaged customers may still exhibit positive outcomes, such as the members of “I hate Facebook” Facebook pages who, ironically, still use the platform (Juric et al., 2015). Meanwhile, a harmful behavioral outcome for other actors is considered as a negative outcome (Juric et al., 2015). These researches believe that CEV can be positive or negative, and it is affected by the direction of engagement (Zhang et al., 2018). There are three main levels of CE—high, medium, and low—in both positive and negative directions (Smith, 2013), and a low negative engagement may yield positive outcomes and vice versa. Signaling theory postulates that the behavior of a customer is based on their interpretation of signals received from the web store. Therefore, this study proposes that:

*Proposition 3(a)*: If the signals provided by the actors are positively interpreted, they are expected to generate positive outcomes such as service co-creation.
*Proposition 3(b)*: If the signals provided by the actors are negatively interpreted, they are expected to generate negative outcomes such as service co-destruction.

## Discussion on Findings

This study responds to the call of recent studies such as Storbacka et al. (2016), Li et al. (2017, 2018) by conceptualizing CEV and identifying the outcomes generated by CEV from the perspective of signaling theory. In this study, CEV is defined as a psychological construct in which web stores generate signals during customer–web store interactions in the form of comments, reviews, star rating, price, information of product, and the esthetic of the website, which are interpreted and acted upon by the customers. Signaling theory explains that the exchange of symmetric knowledge between different actors generates positive outcomes and vice versa (Kirmani and Rao, 2000). Li et al. (2018) aptly define customers’ engagement valence as either positive or negative engagement, but this study proposes that the valence resides in the cognitive interpretation of signals based on signaling theory rather than the outcomes or the brand itself, which is echoed by Hollebeek and Chen (2014), Bowden et al. (2017), and Naumann et al. (2017) and in contrast with Dolan et al. (2017) who claim that valence resides in the outcomes of engagement. This research bases its idea on SDL (Vargo and Lusch, 2008, 2016) and proposes service co-creation and service co-destruction as behavioral outcomes of CEV.

Furthermore, the behavioral outcomes of engagement vary according to the structure of each network. There are two main behavioral outcomes of CEV—positive and negative (Hollebeek and Chen, 2014; Juric et al., 2015)—and customers may share positive (service co-creation) or negative (service co-destruction) experience after engaging with the web store through comments and ratings, which can affect other actors’ decision-making positively (service co-creation) or negatively (service co-destruction; Vargo and Lusch, 2008, 2016).

## Implications, Limitations, and Future Recommendations

Theoretically, this study proposes the outcomes of CEV in an online business context. It is observed that CEV is difficult to operationalize, and observing the direction of customers’ engagement can be tricky. Engagement produces outcomes, and this has yet to be adequately discussed in previous researches, particularly in the context of online service networks such as web stores. Two outcomes are proposed in this study. Positively engaged customers are likely to provide positive comments, supports, and recommendations that are in favor of the brand and participate in service co-creation. Meanwhile, negatively engaged customers are more likely to provide low ratings and negative comments; they engage in service co-destruction. In addition, engagement can be positive, negative, and ambivalent based on symmetric or asymmetric knowledge sharing. Positively interpreted signals may produce positive outcomes (service co-creation), while negatively interpreted signals may produce negative outcomes (service co-destruction).

This framework requires empirical validation to prove the propositions as it is useful for web stores, e-retailers, and customers—web stores can predict the outcomes of the CEV and propose intervention strategies to reduce negative engagement. It should be noted that co-destruction can create a ripple effect affecting many other actors involved in the service network; hence web stores need to minimize the co-destruction probability. In addition, this study raises intriguing questions regarding the nature and extent of the CEV and the measurement of this concept. Since capturing the CEV using subjective measures is not easy due to its psychological state of mind, this study proposes that the CEV should be measured using neuromarketing approaches such as electroencephalogram (EEG) and human-eye tracking (HET). These approaches may generate real-time data that can be useful to predict the outcomes and devise strategies. Also, further research is needed to establish the measurement scales for service co-creation and service co-destruction—Table 2 presents possible propositions and research questions in need of empirical validation. This study is limited as it is conceptual; therefore, empirical validation is needed for more practical implications.

**TABLE 2:** Propositions for research implications.

**Propositions**	**Research question**
P1: Valence of customer engagement lies in the cognitive interpretation of signals sent by other customers and service providers on a web store.	What is the role of service providers in generating positive or negative signals?
P2: Valence of customer engagement shifts between positive, negative, and ambivalent based on the symmetric or asymmetric knowledge sharing or service exchange.	How can information be exchanged symmetrically between different customers in a web store? When is a customer in the state of ambivalent engagement? What are the possible outcomes of ambivalent engagement?
P3a: If the signals provided by the actors are positively interpreted, it is expected to generate positive outcomes such as service co-creation. P3b: If the signals provided by the actors is negatively interpreted, it is expected to generate negative outcomes such as service co-destruction.	What are other possible outcomes of customer engagement valence? In which conditions does customer engagement valence lead to service co-creation and service co-destruction?

## Author Contributions

JS: literature review and write-up. AS: idea generation, concept development, and write-up. IF: concept development. MR: proof of concept. MN: editing and proofreading. All authors contributed to the article and approved the submitted version.

## Conflict of Interest

The authors declare that the research was conducted in the absence of any commercial or financial relationships that could be construed as a potential conflict of interest.
